# Investigating the thin film growth of [Ni(Hvanox)_2_] by microscopic and spectroscopic techniques†

**DOI:** 10.1039/d4na01021c

**Published:** 2025-02-12

**Authors:** Atharva U. Sapre, Jan Vlček, Esther de Prado, Ladislav Fekete, Mariana Klementová, Martin Vondráček, Petr Svora, Emmelyne Cuza, Grace G. Morgan, Jan Honolka, Irina A. Kühne

**Affiliations:** a FZU - Institute of Physics of the Czech Academy of Sciences Na Slovance 1999/2, Prague 8 182 00 Czech Republic kuhne@fzu.cz; b School of Chemistry, University College Dublin Belfield Dublin 4 Ireland; c Czech Technical University in Prague, University Centre for Energy Efficient Buildings Trinecka 1024, Bustehrad 273 43 Czech Republic

## Abstract

We have investigated [Ni(Hvanox)_2_] (H_2_vanox = *o*-vanillinoxime), a square-planar Ni(ii) complex, for the preparation of thin films using organic molecule evaporation. Low pressure experiments to prepare thin films were conducted at temperatures between 120–150 °C and thin films of increasing thicknesses [Ni(Hvanox)_2_] (16–336 nm) have been prepared on various substrates and been analyzed by microscopic and spectroscopic methods. Scanning electron microscopy (SEM), atomic force microscopy (AFM) and transmission electron microscopy (TEM) were used to reveal a rough surface morphology which exhibits a dense arrangement of elongated, rod and needle-like nanocrystals with random orientations. It also enabled us to follow the growth of the thin films by increasing thickness revealing the formation of a seeding layer. X-ray photoelectron spectroscopy (XPS and 3D ED), TEM and X-ray diffraction (XRD) were utilized to confirm the atomic structure and the elemental composition of the thin films.

## Abstract 2

Thin films with thicknesses ranging from 16 to 336 nm of [Ni(Hvanox)_2_] were prepared *via* organic molecule evaporation and analyzed using SEM, AFM, TEM, XPS, and XRD, revealing rough surfaces with nanocrystals, including an initial seeding layer.

## Graphical abstract

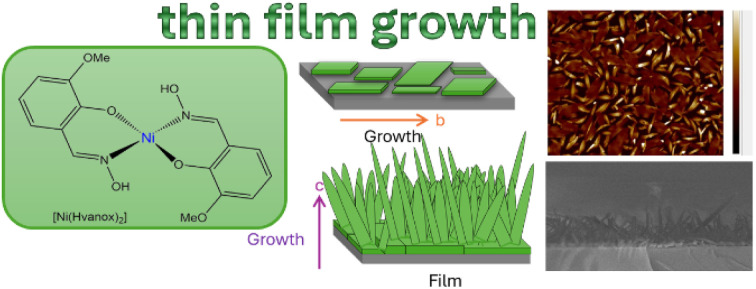

## Introduction

Since Chugaev introduced the gravimetric determination of nickel(ii) in 1905 using its reaction with dimethylglyoxime to produce the characteristic “salmon-red-coloured precipitate” of the [Ni(dmg)_2_] complex,^1^ coordination complexes involving nickel(ii) and oxime ligands have been extensively studied.^2,3^ These complexes are known to exhibit a variety of geometries – octahedral, square planar, or tetrahedral – depending on their coordination environment and the nature of the ligands involved.^4,5^

In recent years, oxime-based nickel(ii) complexes have gained attention for their magnetic properties^5–7^ but also for their versatile roles in catalysis, where they are used in various catalytic reactions like hydrogen evolution,^8^ cross-coupling reactions,^9^ metal-mediated reactions, particularly as intermediates in coupling reactions and radical transformations.^10,11^ More recently, the [Ni(dmg)_2_] complex has been under focus due to its thermal decomposition behaviour and it has been studied extensively as a precursor material to prepare porous and therefore functional nickel oxide nanostructures,^12–14^ with versatile properties leading to applications in fuel cell electrodes, heterogeneous catalysis, and antiferromagnetic layers.^15,16^

Thin films play a critical role in numerous applications, including sensing,^17–20^ catalysis,^21–23^ energy storage,^24–27^ and optoelectronics,^28–30^ where their structural, morphological, and surface properties significantly influence the performance. Among these properties, surface roughness has emerged as a critical parameter in enhancing functional capabilities.^31,32^ Rough surfaces provide increased surface area, promote efficient interaction with surrounding media, and enable unique physical and chemical phenomena not achievable with smoother counterparts. Consequently, the controlled fabrication of thin films with tailored roughness has gained substantial research interest.

This study focuses on the preparation of thin films using the square-planar oxime-based Ni(ii) complex [Ni(Hvanox)_2_] (H_2_vanox = *o*-vanillinoxime). We are exploring the growth dynamics of the thin films and the resulting morphological features. [Ni(Hvanox)_2_] was selected for its square-planar geometry, thermal stability, and resistance to decomposition in air, moisture, or light. The smaller Ni(ii) salicylaldoxime complexes have demonstrated stability up to 200 °C before thermal decomposition.^33,34^

To the best of our knowledge, no studies have investigated [Ni(Hvanox)_2_] or other square-planar Ni(ii) complexes with oxime ligands derived from salicylaldehyde as suitable candidates for thermal evaporation or thin-film preparation *via* vacuum sublimation.

## Results and discussion

### Synthetic procedure

**Scheme 1:**
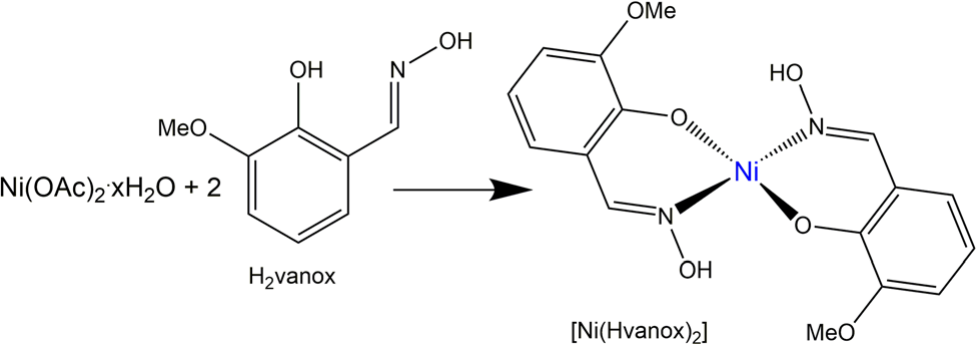
Schematic synthesis of the [Ni(Hvanox)_2_] complex.

[Ni(Hvanox)_2_] was prepared, purified and dried according to the literature procedure,^35,36^ from the reaction of Ni(ii) acetate with the H_2_vanox ligand (*o*-vanillinoxime) according to Scheme 1; for details see Experimental section.

The crystal structure of [Ni(Hvanox)_2_] was reported in the form of a cif by Li *et al.* and was found to crystallize without any solvent in the crystal lattice,^35^ but the structure is well-known since the 1950s.^37,38^

### Thin film preparation

**Fig. 1:**
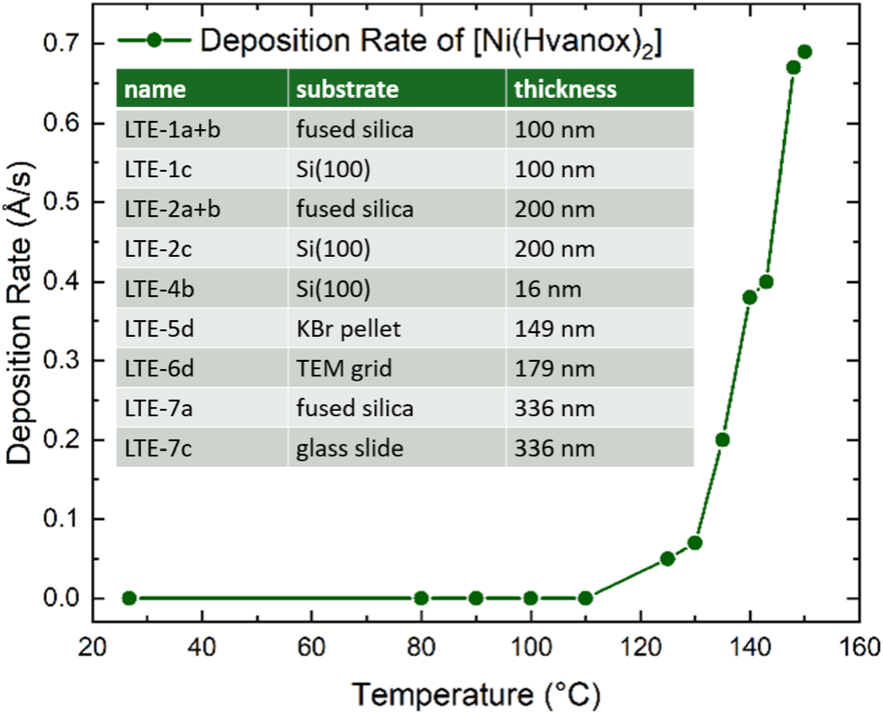
Temperature-dependent deposition rate of [Ni(Hvanox)_2_] at a deposition pressure of 1 × 10^−4^ Pa measured by a QCM sensor; inset: prepared samples including thicknesses.

Thin films of the [Ni(Hvanox)_2_] bulk material were prepared using thermal evaporation under controlled conditions to ensure uniformity and purity. The deposition process was carried out in a vacuum chamber equipped with a Moorfield LTE (Low Temperature Evaporation) source, operating at a process pressure of 1 × 10^−4^ Pa to minimize contamination and ensure high-quality thin films. The evaporation process was controlled by a Lesker FTC-2800C QCM controller in terms of maintaining the deposition rate at 0.3 Å s^−1^. In this setup, the deposition rate is controlled by the power to LTE evaporation source. The dependence between the temperature of the LTE source and the deposition rate was determined during initial experiments (see Fig. 1). A cooled shroud was used during the deposition to suppress unwanted deposition on the chamber walls and to maintain a clean environment for the film growth. We focused on using fused silica and Si(100) as substrates for the thin film deposition. Si(100), due to its atomically flatness, uniform surface, chemical stability and inertness under vacuum and imaging conditions, provides perfect conditions for AFM, SEM and XPS spectroscopy. Moreover, silicon wafers, especially Si(100) are readily available and cost-effective, since they are widely used in the semiconductor industry, making it a great substrate material for low temperature evaporation of molecules. Fused silica on the other hand, shows great thermal stability and chemical resistance which are necessary during the deposition, while the optical transparency (from UV to IR) and surface smoothness are crucial for optical spectroscopy methods. The substrates were positioned 30 cm from the evaporation source and kept at room temperature throughout the process to ensure the as-deposited films adhered well without thermal degradation of the organic molecule. These conditions collectively provided optimal control over the thin-film formation, yielding smooth and reproducible coatings suitable for further characterization.

The samples are named with an integer number between 1 and 7, which each indicates a specific deposition run, leading to thin films with specific thicknesses. The temperature, pressure and the deposition rate of 0.3 Å s^−1^ of all these runs (1–7) were in a similar range and were controlled by the QCM controller. In each run, several substrates (up to 6) were loaded onto the sample stage and were positioned 30 cm from the evaporation source, which were labelled with small alphabetical letters (a, b, c, d, e and f).

### Characterization

#### Infrared and Raman spectroscopy

Infrared (IR) and Raman spectroscopy were used as techniques for characterizing both the bulk starting material, [Ni(Hvanox)_2_], and the prepared thin films. Both methods provide insights into the molecular composition, together with structural information and phase identification.

The IR spectra were recorded on the thin films deposited on KBr pellets and the Raman spectra on thin films deposited on fused silica which are showing the same features as the bulk material, see Fig. S1 and S2† highlighting the successful deposition of the [Ni(Hvanox)_2_] complex by vacuum deposition. The details and band assignment can be found in the ESI.†

#### UV-vis absorbance spectroscopy and optical band gap

**Fig. 2:**
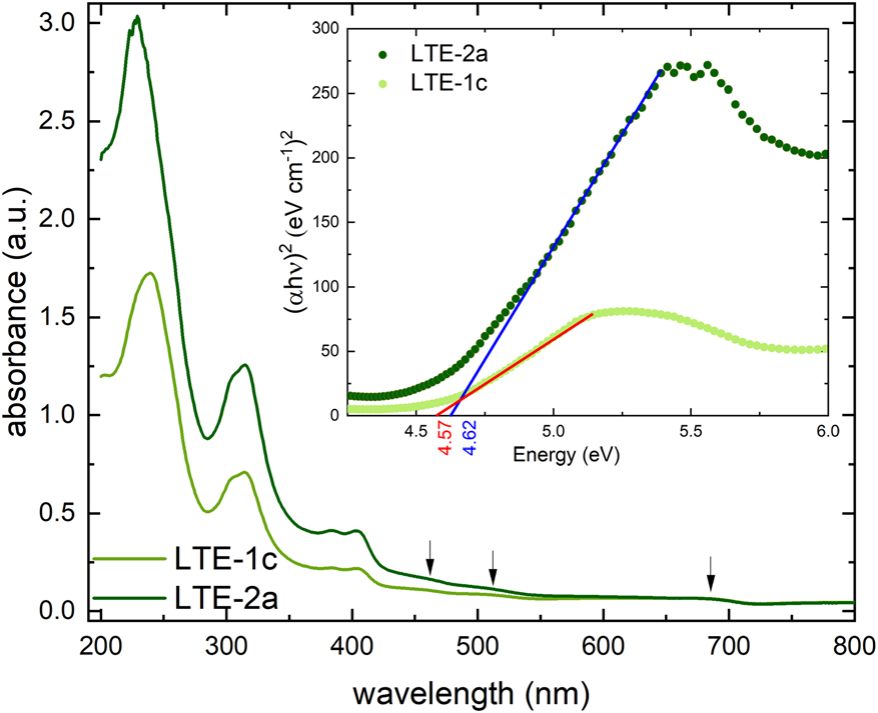
Solid state electronic absorbance spectra of the 100 nm (pale green) and 200 nm (dark green) thin films of [Ni(Hvanox_2_)] deposited on fused silica recorded between 800–190 nm. (d–d-transitions highlighted by arrows); inset: determination of the optical band gap for both thin films.

The absorbance spectra of the 100 nm and 200 nm thin films deposited on fused silica are shown in Fig. 2 and display a strong absorption band at *λ*_max_ = 239 nm for the 100 nm thin film (LTE-1c), which shifts slightly to *λ*_max_ = 230 nm for the 200 nm thin film (LTE-2a). The absorption bands at 313 nm appear for both thin films at the same wavelength. Both absorption bands are assigned as MLCT (metal to ligand charge transfer) bands. The additional intense absorption band at 404 nm and its shoulder at 387 nm are assigned to a phenolate → Ni(ii) charge transfer,^39^ which are also present in the absorbance spectrum of the bulk [Ni(Hvanox)_2_] material (see Fig. S3†).

In square-planar Ni(ii) complexes, the allowed d–d-transitions are between the ^1^A_1g_ ground state and the ^1^A_2g_, ^1^B_1g_ and ^1^E_g_ excited states,^40–42^ which are usually very weak and can be observed in the range between 400–1000 nm (10 000–26 000 cm^−1^).

The solid-state electronic absorbance spectra of the thin films of [Ni(Hvanox)_2_] exhibit only three very weak shoulder-like bands at 460 nm (^1^E_g_ ← ^1^A_1g_), 517 nm (^1^B_1g_ ← ^1^A_1g_), and 680 nm (^1^A_2g_ ← ^1^A_1g_). They are assigned to the three d–d-transitions, respectively, assuming a square-planar environment around Ni(ii).^40–42^

The optical band gap was determined for both thin films and was calculated to be 4.57 eV for the 100 nm thin film and 4.62 eV for the 200 nm thin film, respectively (see Fig. 2, inset). The very small difference of 0.05 eV in the band gap can be due to the change in thickness of the films, since even small differences in morphology like grain size, surface roughness or the appearance/absence of defects can subtly impact the electronic band structure, which can lead to small differences in the band gap.

#### X-ray diffraction analysis

**Fig. 3:**
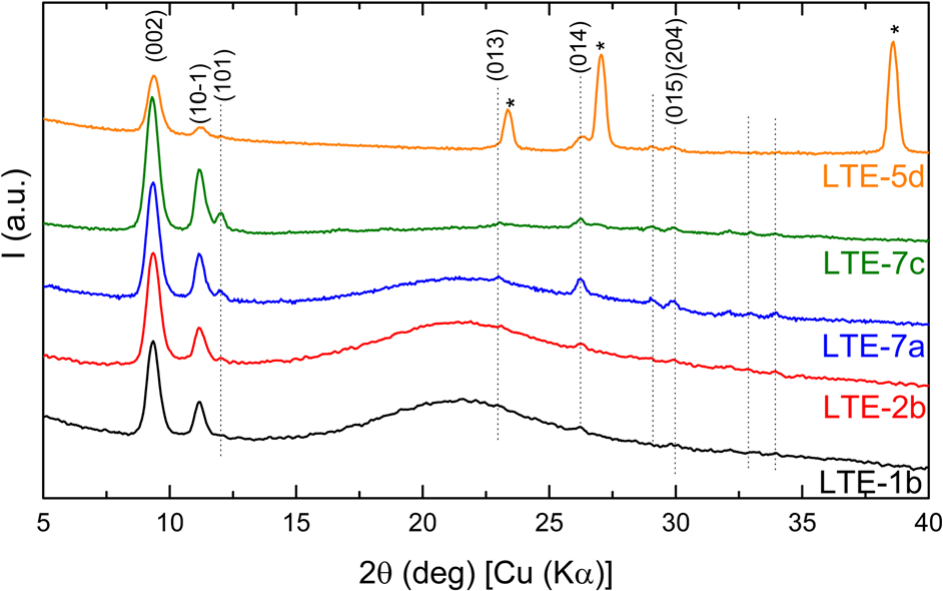
*θ*/2*θ* scans of the sample set, displayed on a quadratic scale, with patterns vertically shifted for clarity. Peaks are indexed by their (*hkl*) indices, except for those generated by the convolution of three or more reflections. Peaks corresponding to the KBr phase are marked with a star. The dotted vertical lines are a visual aid.

The structure and microstructure of the films were investigated through X-ray Diffraction (XRD). Fig. 3 presents the diffraction patterns for the sample set. Only one phase has been detected in the different layers which matches the monoclinic (*P*2_1_/*n*) [Ni(Hvanox)_2_] structure reported by Li *et al.*^35^

All samples exhibit a strong preferred orientation along the [002], [10−1] and [101] crystallographic directions compared to a purely polycrystalline sample. This result is consistent with the morphology observed in the rods-/plate-like crystallites shown in the AFM and SEM images and it is consistent across all samples, regardless of the substrate used. The only difference between the analyzed samples lies in the intensity of the diffraction peaks for Bragg angles greater than 15°. This is because, for thin layers deposited on fused silica (patterns in black and red in Fig. 3), the peaks are barely visible due to the intense halo (15–27°) originating from the amorphous substrate.

**Fig. 4:**
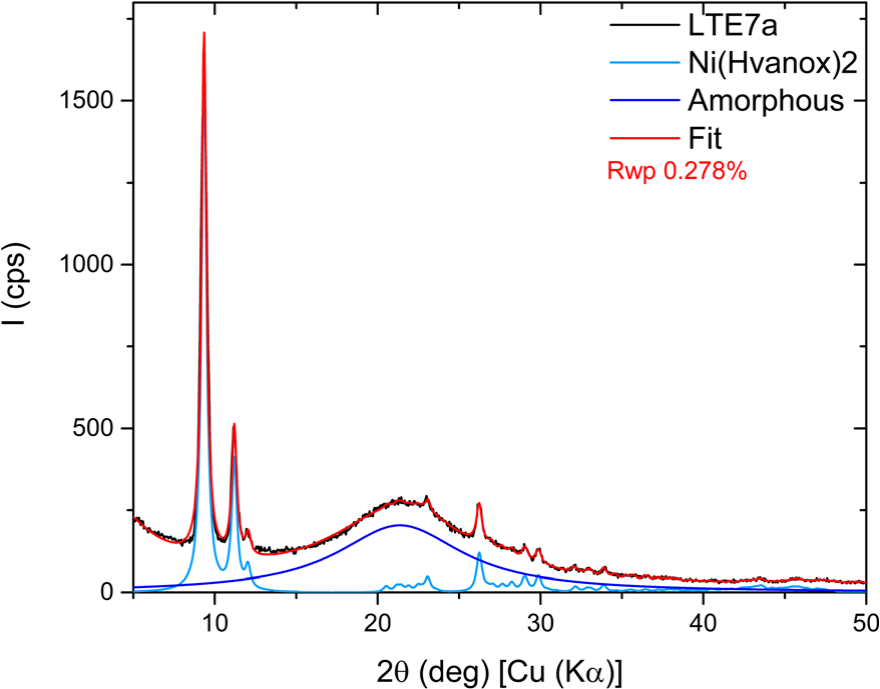
Rietveld refinement results for the LTE-7a sample: the measured data (black), the fit (red), and the contributions of the amorphous (in dark blue) and crystalline (in pale blue) phases.

Since LTE-7a exhibited the highest intensity of the diffraction peaks, it was selected for further detailed analysis. Fig. 4 presents the Rietveld refinement results for that sample: the experimental measured data in black, the fit in red, and the contributions of the amorphous and crystalline phases in dark blue and pale blue, respectively. The refinement results were accurate for peak positions but showed discrepancies in intensity. Due to the extremely low intensity and the presence of three preferred orientations, the Pawley approach was then included during the refinement. This process involved treating the intensity as a free parameter to be refined rather than calculating its contribution from the atomic positions. The refinement improved significantly, achieving a figure of merit Rwp = 0.278%. The refined lattice parameter values are *a* = 8.396 Å, *b* = 4.862 Å, *c* = 19.181 Å, and *β* = 95.76°. Given that the diffraction peaks of all five samples are almost perfectly aligned, it is assumed that their lattice parameters will be highly similar.

The structure was also confirmed by 3D electron diffraction (3D ED) on several single crystals (see below).

#### Atomic force microscopy

**Fig. 5:**
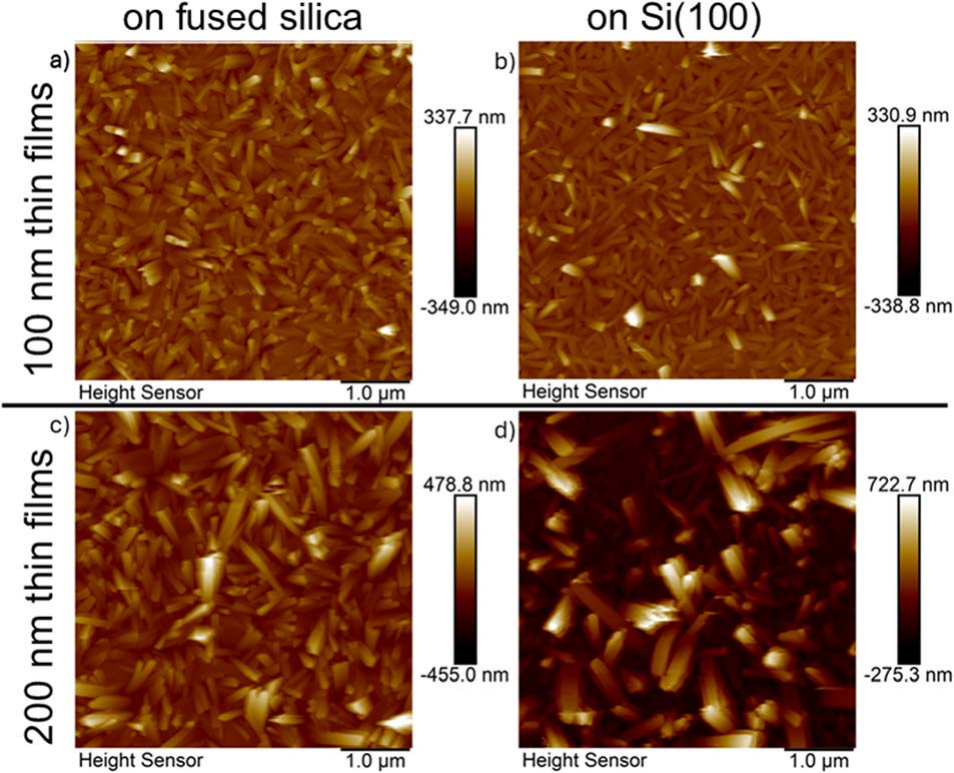
AFM micrographs (area: 5 μm × 5 μm) of the 100 nm thin films (top; a and b) and 200 nm thin films (bottom; c and d) deposited on fused silica (left; a and c) and on Si(100) (right; b and d) using a lateral resolution of 512 × 512 points^2^.

The surface morphology of the thin films with increasing thicknesses was examined using atomic force microscopy (AFM). The AFM micrographs of the thin films LTE-1a and LTE-1c (100 nm) and LTE-2a and LTE-2c (200 nm) of the [Ni(Hvanox)_2_] deposited on fused silica and Si(100) are shown in Fig. 5.

The morphology of the thin films exhibits in all cases a dense arrangement of elongated, rod and needle-like nanocrystals with random orientations. The average length in the 100 nm thin films varies between 300–400 nm but can even go up to 600 nm in length with an average width of 40 nm. By increasing the thickness of the films to 200 nm, the length of the nanocrystals increases but interestingly the width almost doubles to 60–90 nm in average when deposited on fused silica, but significantly increases up to an average width of 130–170 nm when deposited on Si(100) (see Fig. 5).

The height indicator spans over 700–1000 nm, indicating significant variation in the topography. The texture of the surface appears highly uneven with the surface roughness (RMS) values of 40.6 nm (79.8 nm) for the 100 nm (200 nm) thin film deposited on fused silica and 39.2 nm (155 nm) for the 100 nm (200 nm) thin film deposited on Si(100), respectively, indicating that there is hardly any difference in the 100 nm thin film on the choice of substrate, but once thicker films are deposited, a rougher surface is observed when deposited on Si(100). The difference in the nanocrystal width deposited on Si(100) and fused silica substrates can arise due to the distinct properties of these substrates like surface roughness, thermal conductivity/capacity or surface charge effects, which can all influence the nucleation, crystal growth, and film morphology. Si(100) typically has a smoother surface compared to fused silica and can therefore promote more uniform nanocrystals. In addition, Si(100) also has higher thermal conductivity which allows heat to be dissipated more efficiently during the deposition process. This promotes steady crystallization and grain growth, resulting in larger and more well-defined crystals.

**Fig. 6:**
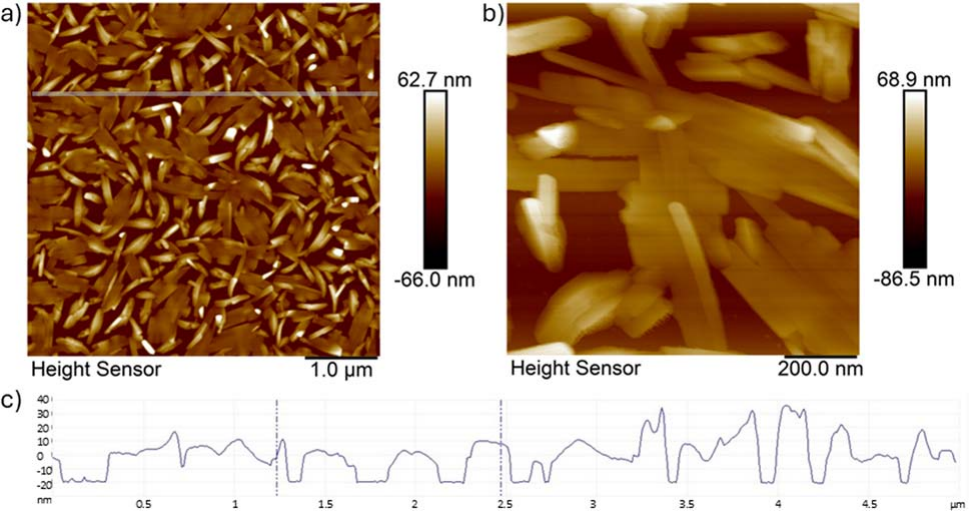
AFM micrographs of the 16 nm thin film (top; a and b) deposited on Si(100) using different magnifications (a) and (b); the scanned area in (a): 5 μm × 5 μm with a lateral resolution of 512 × 512 points^2^; (bottom; c) the height indicator profile along the line indicated in (a).

In order to investigate the growth of the nanocrystals on the substrate, very thin films of 16 nm were deposited on Si(100) and analysed using AFM microscopy (see Fig. 6).

The images in Fig. 6 indicate that the first layer of crystals which are formed on the substrate surface are aligned flat with rod-shaped crystals even aligning parallel to form crystalline “patches”. The crystals already show the preferred elongation in one direction with lengths varying between 400–600 nm and a width of 30–40 nm. This first layer most likely acts as a seeding layer on the substrate, where the next needle-like crystals will grow perpendicular on top of that.

#### X-ray photoelectron spectroscopy

In order to avoid charging of the sample, a very thin film of 16 nm (LTE-4b) was deposited on a silicon Si(100) substrate for XPS spectroscopy. This thin film sample is thin enough and is not charging more than 1 eV, and as observed from the AFM images, the thin film is not continuous, and therefore the substrate is still visible in the XPS spectra.

**Fig. 7:**
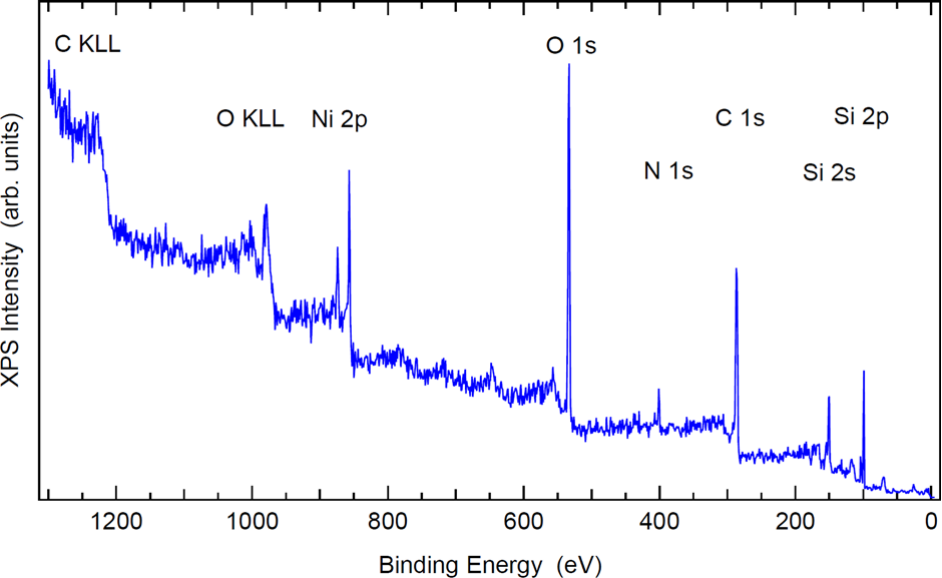
XPS survey spectrum of the 16 nm thin film deposited on Si(100) with principal photoelectron and Auger peaks labelled.

**Fig. 8:**
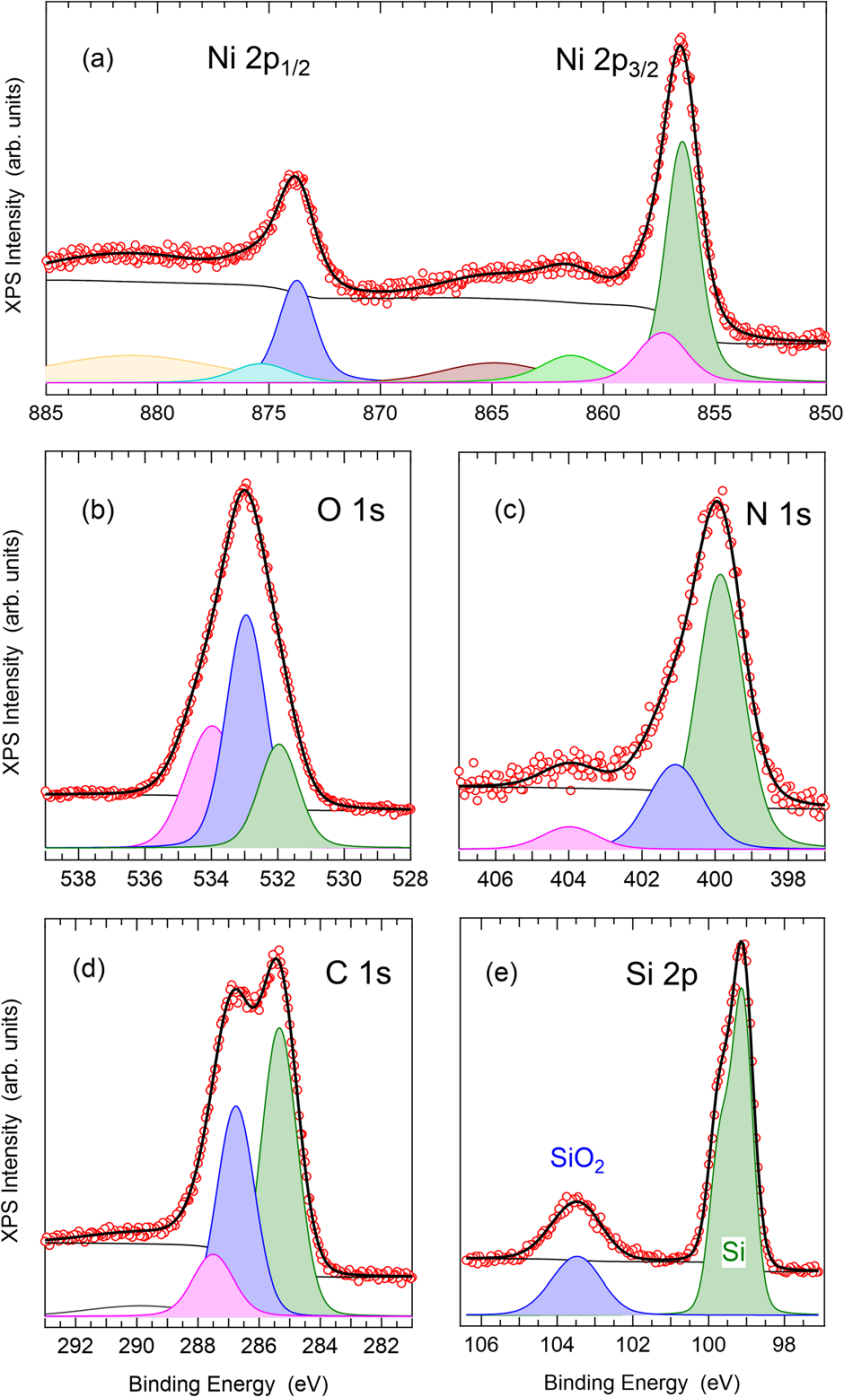
High resolution XPS core level spectra of the 16 nm thin film deposited on Si(100) of Ni 2p (a), O 1s (b), N 1s (c), C 1s (d), and Si 2p (e). Experimental data (open circles) were fitted (thick curve) with Voigt peaks (filled peaks) on Shirley background (thin curve).

The XPS spectra, see Fig. 7 and 8, reveal a chemical composition of 4.5% of nickel,^43^ 24% of oxygen, 7.5% of nitrogen and 64% of carbon, which is in good agreement with the chemical composition of [Ni(Hvanox)_2_]. The elemental composition is based on the XPS peak areas normalized to the photoionization cross section. The silicon peaks with lower binding energy originate from the substrate wafer, whereas those with +4 eV energy shift are from native oxide. The highest binding energy O 1s peak is also from native silicon oxide.^44^

The lower BE (binding energy) C 1s peak belongs to the sp^2^ C–C and C–H bonds from the aromatic ring as well as the sp^3^ C–H from the methoxy group, respectively, while the higher BE peak arises from the C–O bonds.^45^ The C=N peak is most likely the highest energy peak. The fitting model has peak areas exactly in the ratio 4 : 3 : 1 (green : blue : magenta peak in Fig. 8) in good agreement with the singly deprotonated Hvanox^−^ ligand.

The nickel Ni 2p XPS spectrum can be compared with other Ni(ii) complexes in the literature,^46,47^ and shows the main characteristic peaks of a Ni(ii) species at 856 eV and 874 eV which can be assigned to Ni 2p_3/2_ and Ni 2p_1/2_, respectively, and are in a similar range as observed for other square-planar Ni(ii) complexes with oxime-based ligands.^48,49^ A multiplet splitting is not expected for a low spin Ni(ii) compound with a spin ground state of *S* = 0.^50^ The diamagnetic nature of the bulk material was confirmed by magnetic measurements, and the results are shown in the Experimental section and Table 1.

**Table 1:** Temperature dependent *χ*_M_*T* values for the bulk [Ni(Hvanox)_2_] complex

*T* (K)	*χ*_M_ *T* (cm^3^ K mol^−1^)	*T* (K)	χ_M_*T* (cm^3^ K mol^−1^)
300.01	0.06	100.02	0.02
200.02	0.04	20.00	0.01

The interpretation of the O 1s and N 1s positions are in accordance with other salen-type transition metal complexes.^51,52^

#### Scanning electron microscopy

**Fig. 9:**
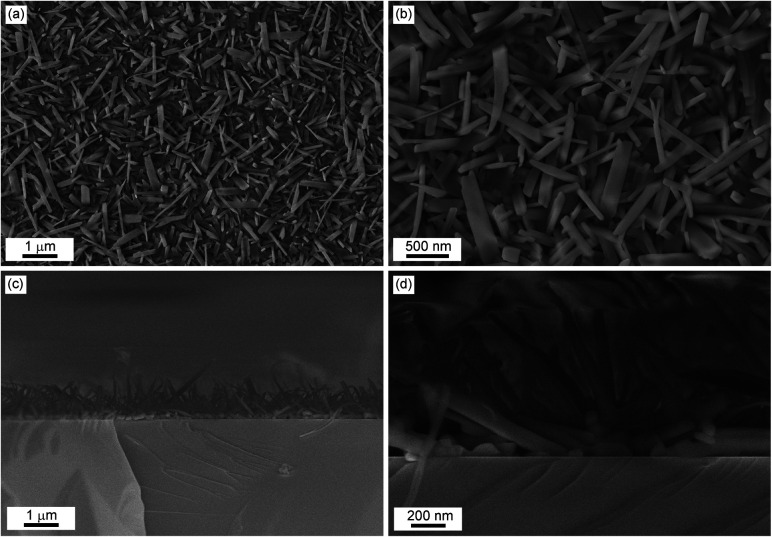
Top: Representative SEM images (secondary electrons) of a 200 nm [Ni(Hvanox)_2_] film deposited on Si(100) with different magnifications: (a) and (b); Bottom: cross-section view using different magnifications: (c) and (d).

The microstructure and surface morphology of the [Ni(Hvanox)_2_] film with a thickness of 200 nm, deposited on a silicon Si(100) substrate (LTE-2c) to avoid charging, are shown in the scanning electron microscopy (SEM) images in Fig. 9.

The SEM images show that the surface texture is rough, and the entire substrate is covered with needle-like crystals (see also Fig. S4, ESI†), which on average exhibit a length between 400 nm–1 μm. In some cases, they can reach even up to 2 μm length, with a diameter of 70–120 nm, as highlighted in Fig. 9. The microcrystals of the [Ni(Hvanox)_2_] film arrange randomly on the surface and a high degree of structural uniformity is demonstrated by the distribution of the microcrystals across the substrate.

The cross-section view, Fig. 9, reveals that the nanocrystals exhibit a unique growth dynamic with a seeding layer as the first layer on the substrate followed by the growth of needle-like crystals perpendicular to that. The seeding layer, as shown in the SEM image of the cross section, exhibits a thickness of 70–150 nm, which is in good agreement with the AFM results of the very thin film. On top of the seeding layer, the nanocrystals grow perpendicular to the surface of the substrate and range in length between 600 nm to 1.5 μm. Despite this perpendicular growth orientation, the alignment of the nanocrystals is random in nature. This vertical growth orientation is most likely driven by the minimization of the surface energy.

#### Transmission electron microscopy and electron diffraction

TEM and STEM images were first recorded on a thin film deposited directly onto a holey-carbon TEM grid, LTE-6d. The results show that the crystals align with the TEM grid and adapt its structure, leading to a “wavey” pattern, which in addition exhibits circular holes, see Fig. S5, ESI.† The holes arise from the non-continuity of the carbon film on the TEM grid, and places where a seeding layer cannot be deposited, and therefore the thin films exhibit defects in the form of holes. STEM images of the same sample show a dense array of elongated, rod-like nanostructures. These nanostructures are uniformly distributed and oriented in various directions, giving the surface a textured appearance that appears to grow in “waves”, see Fig. S6, ESI.† The images at higher magnification highlight that usually between 2–4 neighbouring nano-rods align in the same direction, leading to a more “oriented” film than when deposited on fused silica or Si(100) which were used for the SEM and AFM measurements.

**Fig. 10:**
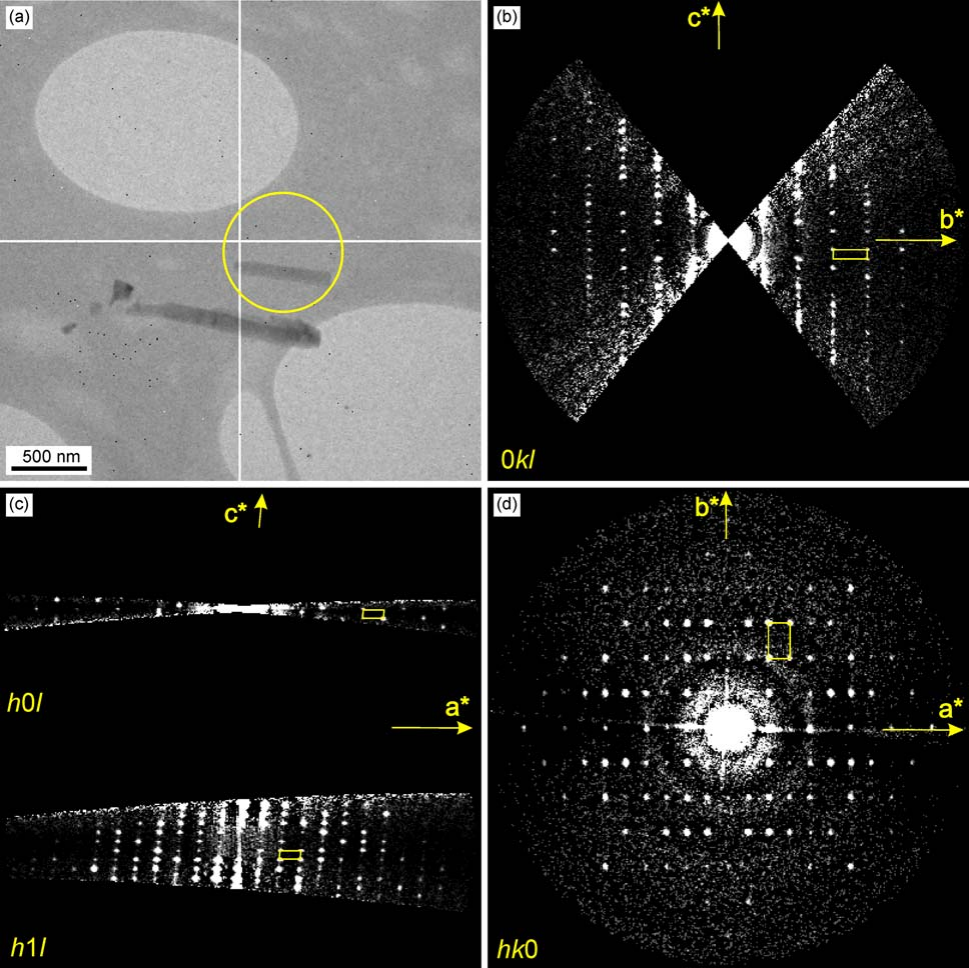
Electron diffraction results. (a) Image of crystals (crystal used for 3D ED experiment is shown in the yellow circle), (b) 0*kl* section through the 3D ED data set, (b) *h*0*l* and *h*1*l* sections through the 3D ED data set documenting the extinction rule for *n*-glide plane perpendicular to *b*-axis (*h* + *l* = 2*n* + 1), (c) *hk*0 section through the 3D ED data set. Yellow parallelepiped shows the corresponding projection of the unit cell.

In addition, an electron diffraction experiment was performed using some nanocrystals scraped off from the thick film sample LTE-7a from the XRD experiment (Fig. 10). The nanocrystals are quite beam sensitive, therefore the diffraction experiment was performed while cooling the sample to 90 °C with liquid nitrogen. Reciprocal space sections of the 0*kl*, 1*kl*, *h*0*l*, *h*1*l*, *hk*0 and *hk*1 planes were reconstructed from the 3D data set collected by continuous rotation from ±50°, shown in Fig. 10. The sections clearly show individual spots confirming the crystallinity of the material. The 3D ED data were even good enough, despite the low data completeness (53% for a monoclinic structure), to solve the structure *ab initio* by the chargeflipping method^53^ and refine it using the kinematical approach, perfectly matching the structure of [Ni(Hvanox)_2_] by Li *et al.*,^35^ with *R*-values of: *R*(obs) = 17.08%, *wR*(obs) = 23.49%, *R*(all) = 24.19% and *wR*(all) = 26.48%, GOF(obs) = 2.63, GOF(all) = 1.95.

## Conclusions

In this study we have focused on the preparation and analysis of thin films of [Ni(Hvanox)_2_] with thicknesses ranging from 16 to 336 nm, deposited primarily on fused silica and Si(100) substrates. Optical and spectroscopic techniques were used to analyse the thin films. X-ray photoelectron spectroscopy (XPS) confirmed the expected chemical composition and high purity, using the XPS core level spectra of Ni 2p, O 1s, N 1s and C 1s, while also confirming the square-planar arrangement of the Ni(ii) centre. Powder X-ray diffraction (XRD) indicated phase purity, which is in good agreement with the theoretical pattern. 3D electron diffraction *via* TEM on single nanocrystals confirmed the monoclinic crystal structure corresponding to the known structure of [Ni(Hvanox)_2_].

Scanning electron microscopy (SEM), atomic force microscopy (AFM) and transmission electron microscopy (TEM) analyses reveal rough surface morphology in the thin films, characterized by densely packed, randomly oriented rod- and needle-like nanocrystals. SEM and AFM confirm a two-stage growth dynamic: a 70–150 nm thick seeding layer forms on the substrate, followed by the perpendicular growth of needle-like crystals. AFM shows uneven surface textures with RMS roughness values of 40.6 nm (79.8 nm) for 100 nm (200 nm) films on fused silica and 39.2 nm (155 nm) for 100 nm (200 nm) films on Si(100). The substrate choice has minimal impact on 100 nm films, but thicker films exhibit greater roughness on Si(100).

These very rough, thin films of [Ni(Hvanox)_2_] have diverse potential for future applications, leveraging their unique surface properties, high surface area, and nanostructured morphology. We will attempt to utilize them as chemical sensors in the next step, where the increased surface area has the potential to enhance the interactions with target molecules, and lead to improved sensitivity. Additionally, they also hold promise in heterogeneous catalysis, offering more active sites for reactions, and in electrocatalysis for energy storage and conversion applications, such as in fuel cells and batteries.

## Experimental

### Materials and methods

#### Starting materials

The fused silica and Si(100) substrates were cleaned before deposition in an ultrasonic bath in isopropanol for 10 minutes, to remove any organic impurities on the surface, followed by drying in a nitrogen atmosphere. The Si(100) substrates were used as such and were not treated chemically to remove the passivation oxide layer which is usually apparent on the Si(100).

All chemicals and solvents if not otherwise mentioned were purchased from chemical companies and were reagent grade. They were used without further purification or drying. All reactions were carried out under ambient conditions. All measurements were carried out on powdered samples of the respective polycrystalline compound.

#### Synthesis of [Ni(Hvanox)_2_]

The synthesis of [Ni(Hvanox)_2_] was done according to the literature:^35,36^ 2.0 mmol of H_2_vanox ligand (*o*-vanillinoxime (H_2_vanox) was prepared according to the procedures described in the literature^54,55^) were dissolved in 20 mL ethanol, 1.0 mmol Ni(ii) acetate hydrate was added, and the suspension was stirred for 6 hours. After that, the green product was filtered and washed several times with ethanol and water to remove any remaining starting materials and dried in air. (Yield average: 327 mg, 83.7%)

Elemental analysis for [NiC_16_H_16_N_2_O_6_] (%). Calculated: C: 49.15, H: 4.12, N: 7.16; found: C: 49.22, H: 4.03, N: 7.19.

IR (KBr): *ν̃* = 3365 w, 2931 s, 2838 m, 2359 w, 1644 m, 1601 s, 1555 s, 1511 s, 1470 s, 1454 s, 1384 w, 1359 s, 1333 s, 1298 s, 15′252 s, 1225 s, 1197 m, 1168 w, 1103 m, 1080 m, 1023 s, 977 s, 946, 875 m, 776 m, 763 m, 728 s, 638 m, 577 w, 547 w, 428 w.

Magnetic measurements using a SQUID magnetometer confirmed the square-planar environment. The magnetic moment was measured in a temperature range between 300–20 K and the sample was found to be diamagnetic over the entire temperature range, see Table 1.

#### Physical measurements

All measurements were carried out on both, powdered samples of the polycrystalline compound, and the prepared thin films.

#### Elemental analysis

(C, H and N) was performed using a Vario EL (Elementar Analysen System GmbH) from PerkinElmer. Fourier transform infrared spectra (FT-IR) were recorded in the form of KBr pellets (or as thin films deposited on KBr pellets) on a Bruker Invenio S in the range between 4000–400 cm^−1^. The Raman spectra of the bulk material and thin films were recorded using a Renishaw InVia™ Raman spectrometer with a laser excitation wavelength of 488 nm. Baseline correction, smoothing, and peak identification were applied to the spectra using the Renishaw WiRE software.

Solid-state UV-vis spectra were recorded on an Agilent Cary 60 UV-vis spectrometer. Solid state samples were prepared for UV-vis by grinding a small amount of bulk material (*ca* 1 mg) into 130 mg of KBr, which was pressed into a disk (15 mm diameter) under 12.5 tonnes pressure for 90 seconds. The spectrum of this solid-state disc was then the recorded using the solid-state sample holder. The spectrum of the bulk [Ni(Hvanox)_2_] material is shown in Fig. S3, ESI.† The solid-state UV-vis spectra of the thin films deposited on fused silica were recorded as such using a solid-state sample holder.

The magnetic susceptibility measurements were recorded on a Quantum Design SQUID magnetometer (MPMS-XL) operating between 1.8 and 300 K. DC measurements were performed on the bulk polycrystalline sample only. The sample was wrapped in a gelatine capsule without constraining.

X-ray diffraction (XRD) measurements were performed using Cu Kα radiation in a SmartLab SE Multipurpose Rigaku diffractometer (Rigaku Corporation, Tokyo, Japan) with a HyPix-3000 2D detector operating in 0D mode and using parallel beam geometry. Patterns were collected in the range (5–100°) using the grazing-incidence configuration, being the incident angle *ω* between 0.3° and 0.5°, depending on the sample. The optics in the incident beam consisted of a multilayer parabolic mirror, 0.25 mm incident slit and a 2.5° Soller slit. In the case of the diffracted beam, the optics consisted of a 0.5° parallel plate analyser and a 2.5° Soller slit. Additionally, a beam knife was mounted on the diffraction arm to enhance the signal-to-noise ratio by reducing unwanted surface scattering. Phase identification was performed using SmartLab Studio II software with access to the PDF-5 database, while Rietveld refinements were carried out using TOPAS V3 software.

AFM images of the thin films were recorded at room temperature on an ambient AFM (Bruker, Dimension Icon) in Peak Force Tapping mode with ScanAsyst Air tips (Bruker; *k* = 0.4 N/m; nominal tip radius 2 nm). The measured topographies have 512 × 512 points (ref. 2) resolution. The SEM images were recorded using a Scanning electron microscope Zeiss Merlin with low high voltage (3 kV) and low current (100 pA).

Transmission electron microscopy (TEM) was carried out on an FEI Tecnai TF20 X-twin microscope operated at 200 kV (FEG, 2.5 Å point resolution). The microscope was also used in scanning mode (STEM) with a High-Angle Annular Dark Field Detector (HAADF). TEM images were recorded on a Gatan UltraScan CCD camera with resolution of 2048 × 2048 pixels using the Digital Micrograph software package.

The structure determination of single nanocrystals was performed by 3D electron diffraction (3D ED). The data were collected on a TEM (Tecnai G2 20, FEI) operated at 200 kV with a LaB_6_ cathode, equipped with an ASI Cheetah direct detection camera (512 × 512 pixels), using the continuous-tilt approach (tilt range ±50 deg) at 90 °C. The data were processed in the PETS 2.0 software.^56^ The structure was solved by the Superflip software,^53^ and refined using Jana2020.^57^

Photoemission electron microscope (PEEM) images (see Fig. S7, ESI†) and XPS spectra were recorded on a 16 nm thin layer of [Ni(Hvanox)_2_] on a Si wafer (sample LTE-4b) and the spectra and images were recorded on the NanoESCA device at FZU (Scienta Omicron).

## Data availability

All data (IR/Raman/UV-vis spectra, XRD pattern, microanalysis, XPS spectra, AFM/SEM/TEM images) that support the findings of this study are available within the article and its ESI.† The raw data of this study are available from the corresponding author upon request.

## Author contributions

Conceptualization – I. A. K., J. V.; sample preparation – I. A. K., A. U. S., J. V.; XRD – E. deP.; IR spectroscopy – I. A. K., A. U. S.; Raman spectroscopy – A. U. S.; XPS – M. V., J. H.; AFM – L. F.; SEM – P. S.; TEM – M. K.; UV-vis spectroscopy – J. V., E. C.; magnetic measurements – E. C.; elemental analysis: E. C.; formal analysis – J. V., I. A. K.; funding acquisition – J. V., I. A. K., G. G. M.; project administration – J. V.; resources – I. A. K., J. V.; supervision – J. V., I. A. K., G. G. M.; visualization – I. A. K., L. F., A. U. S., M. V., P. S., E. P.; writing original draft – I. A. K., J. V.; writing, review and editing – all authors. The manuscript was written through contributions of all authors. All authors have given approval to the final version of the manuscript.

## Conflicts of interest

There are no conflicts to declare.

## Supplementary Material

NA-007-D4NA01021C-s001
