# Supplementary material for: Investigating the thin film growth of [Ni(Hvanox)2] by microscopic and spectroscopic techniques
Source: Nanoscale Adv. 2025 Feb 12;7(7):2083–91. doi: 10.1039/d4na01021c (PMC11843256; doi:10.1039/d4na01021c)
Supplement: NA-007-D4NA01021C-s001 [file NA-007-D4NA01021C-s001.pdf]

Supplementary Information (SI) for Nanoscale Advances.
This journal is © The Royal Society of Chemistry 2025

Investigating the thin film growth of [Ni(Hvanox)$_2$] by microscopic and spectroscopic techniques

Atharva U. Sapre,[a] Jan Vlček,[a] Esther de Prado,[a] Ladislav Fekete,[a] Martin Vondráček,[a] Petr Svora,[a] Emmelyne Cuza,[b] Grace G. Morgan,[b] Jan Honolka[a] and Irina A. Kühne*,[a]

Contents

S1 Infrared and Raman Spectroscopy 2

Figure S1. IR spectra of the bulk starting material, [Ni(Hvanox)$_2$] (top, purple) pressed into a KBr pellet compared with the thin films LTE-3c (130 nm) and LTE-5d (150 nm) deposited on KBr pellets. .2

Figure S2. Raman spectra of the bulk starting material, [Ni(Hvanox)$_2$] (top, green) compared with the thin film LTE-2a (200 nm) deposited on fused silica. 3

Figure S3. Solid state electronic absorbance spectra of the bulk [Ni(Hvanox$_2$)] material recorded between 800 – 250 nm. 3

Figure S4. SEM image (secondary electrons) of the surface of a 200 nm thin film of [Ni(Hvanox)$_2$] deposited on a Si(100). 4

S2 TEM Microscopy 5

Figure S5. TEM images of a thin film of [Ni(Hvanox)$_2$] deposited on a holey-carbon TEM grid using different magnifications (sample LTE-6d, thickness 180 nm). 5

Figure S6. STEM images of a thin film of [Ni(Hvanox)$_2$] deposited on a TEM grid using different magnifications (sample LTE-6d, thickness 180 nm). 6

Figure S7. PEEM images of the center of the sample, field of view 102 µm, excited using a Hg-lamp (hν = 5.2 eV, left) and by Al Kα (hν = 1486.7 eV, right) show homogeneous coverage of the substrate with fine grains. 7

S3 References 7

S1 Infrared and Raman Spectroscopy

The IR spectra were recorded on the thin films deposited on KBr pellets which are showing the same features as the bulk material, see Figure S1 highlighting the successful deposition of the [Ni(Hvanox)$_2$] complex.

The rather broad bands at 3440 cm^{-1} are assigned to the O-H stretching in the vanillinoxime ligand. The presence of these broad O-H bands often appear together with two other bands at 2360 cm^{-1} and 2340 cm^{-1} in metal chelates using oximes based on salicylaldehyde which point out to the presence of strong intramolecular hydrogen bonding.[1]

The bands at 1646 cm^{-1} and 1602 cm^{-1} are indicative of the characteristic absorption bands of the imine part, C=N, of the oxime group and the aromatic C=C stretching, respectively. The phenolic C-O stretching is assigned to the bands at 1512 cm^{-1}, and the oxime N-O band at 1225 cm^{-1}. All bands are found in the thin films as well as in the bulk material.

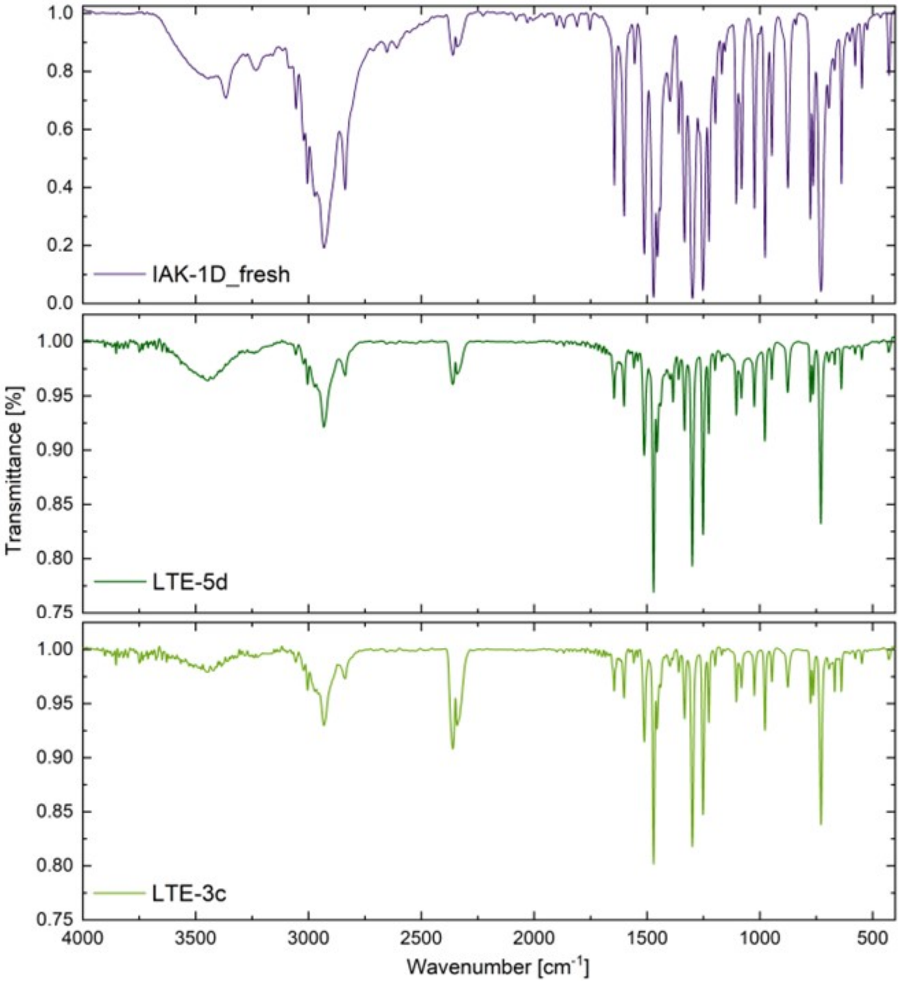

Figure S1. IR spectra of the bulk starting material, [Ni(Hvanox)$_2$] (top, purple) pressed into a KBr pellet compared with the thin films LTE-3c (130 nm) and LTE-5d (150 nm) deposited on KBr pellets.

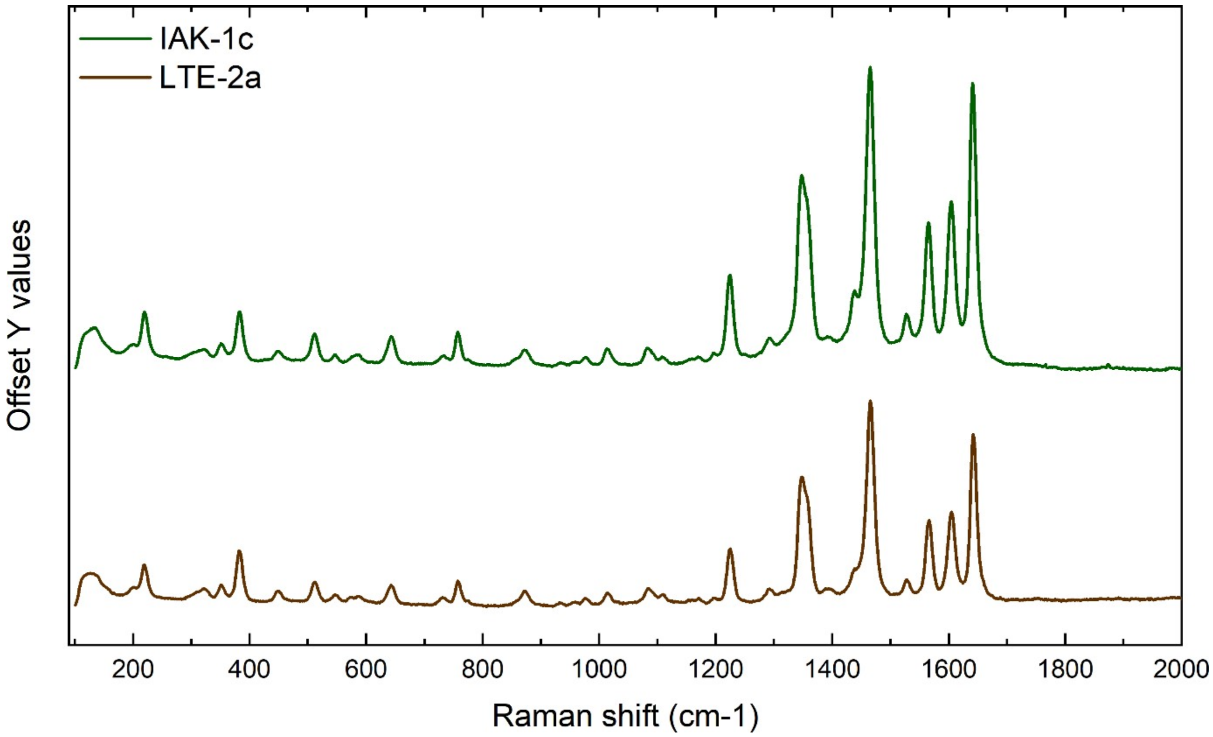

Figure S2. Raman spectra of the bulk starting material, [Ni(Hvanox)$_2$] (top, green) compared with the thin film LTE-2a (200 nm) deposited on fused silica.

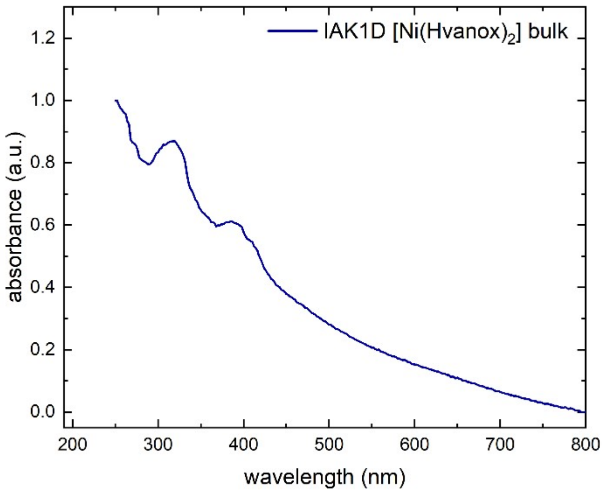

Figure S3. Solid state electronic absorbance spectra of the bulk [Ni(Hvanox$_2$)] material recorded between 800 – 250 nm.

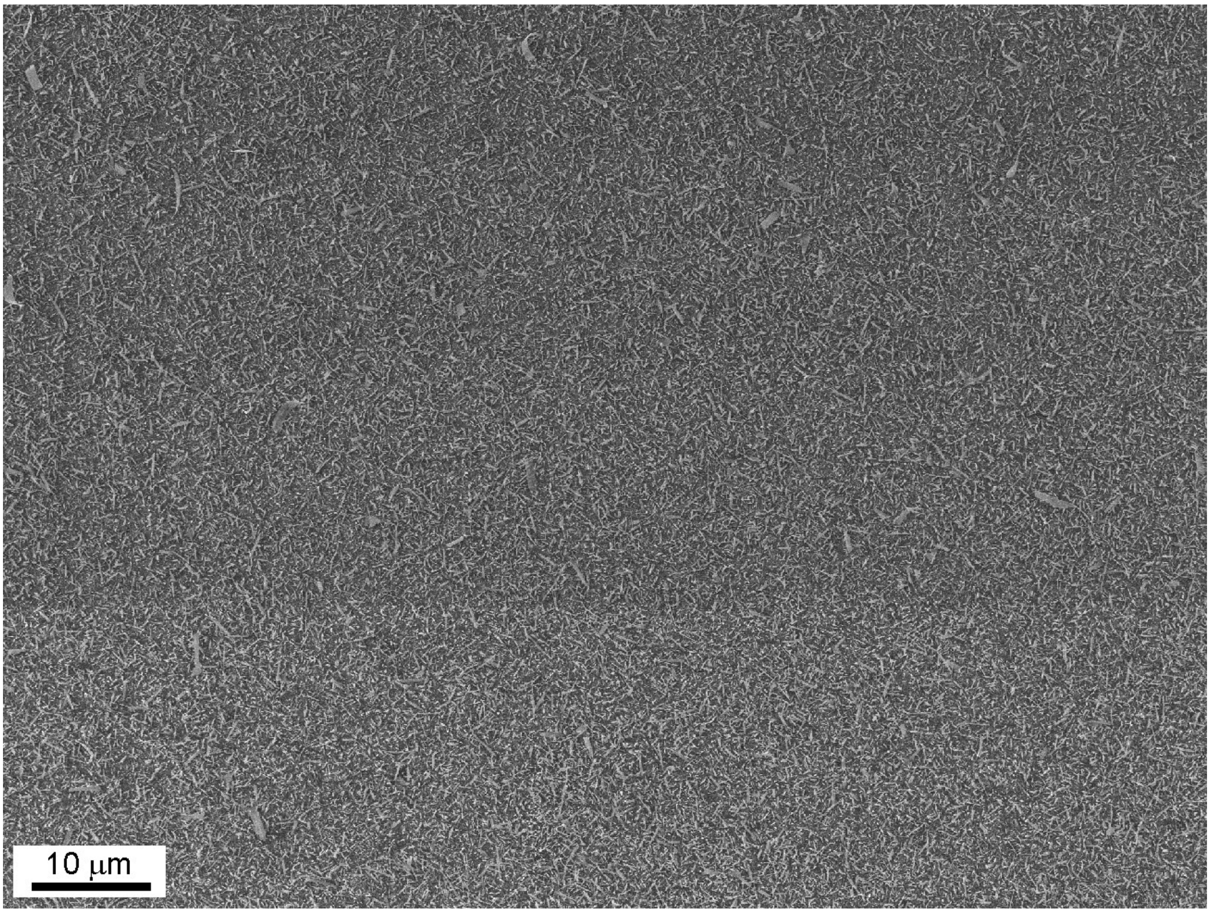

Figure S4. SEM image (secondary electrons) of the surface of a 200 nm thin film of [Ni(Hvanox)$_2$] deposited on a Si(100).

S2 TEM Microscopy

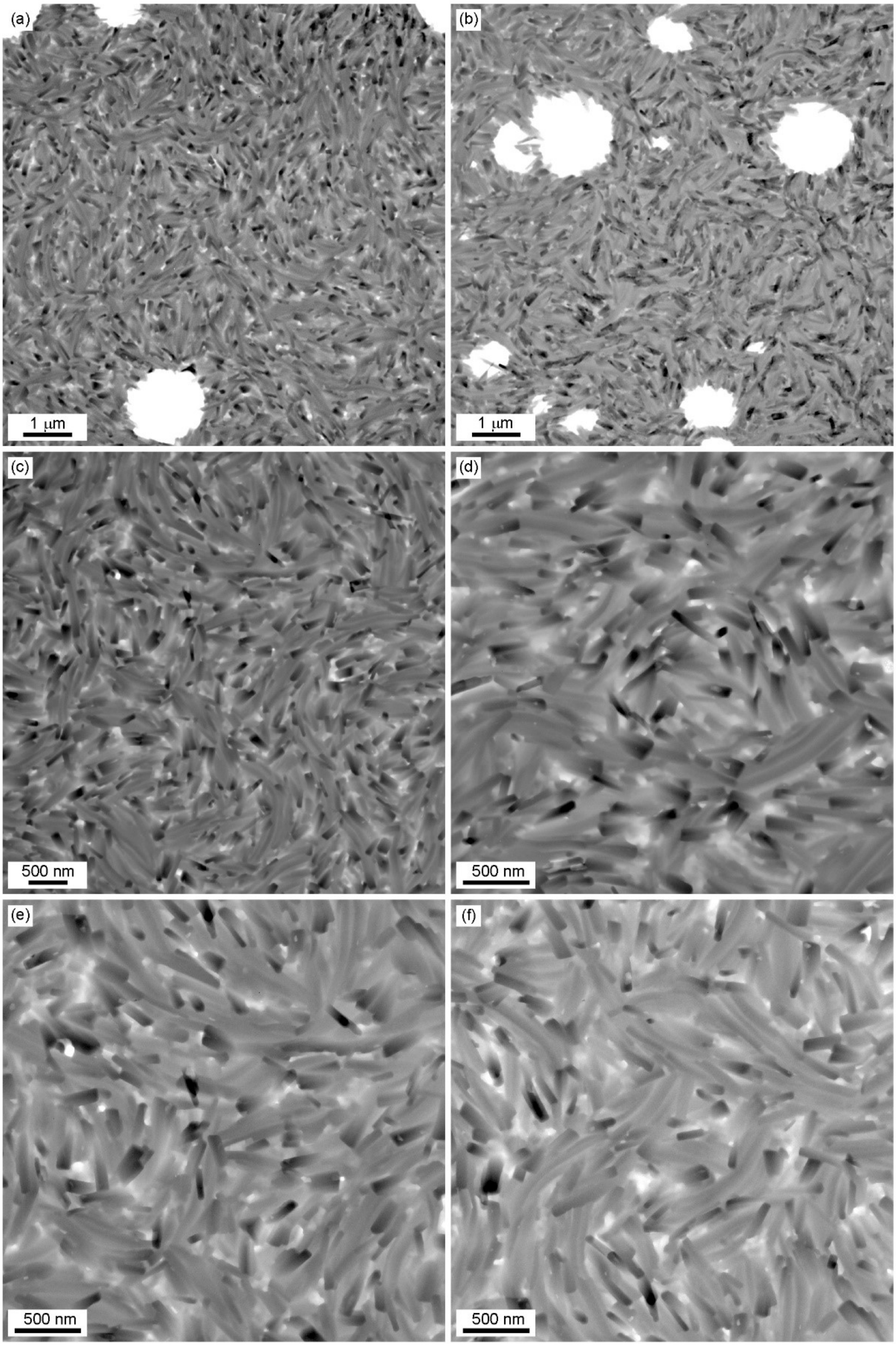

Figure S5. TEM images of a thin film of [Ni(Hvanox)$_2$] deposited on a holey-carbon TEM grid using different magnifications (sample LTE-6d, thickness 180 nm).

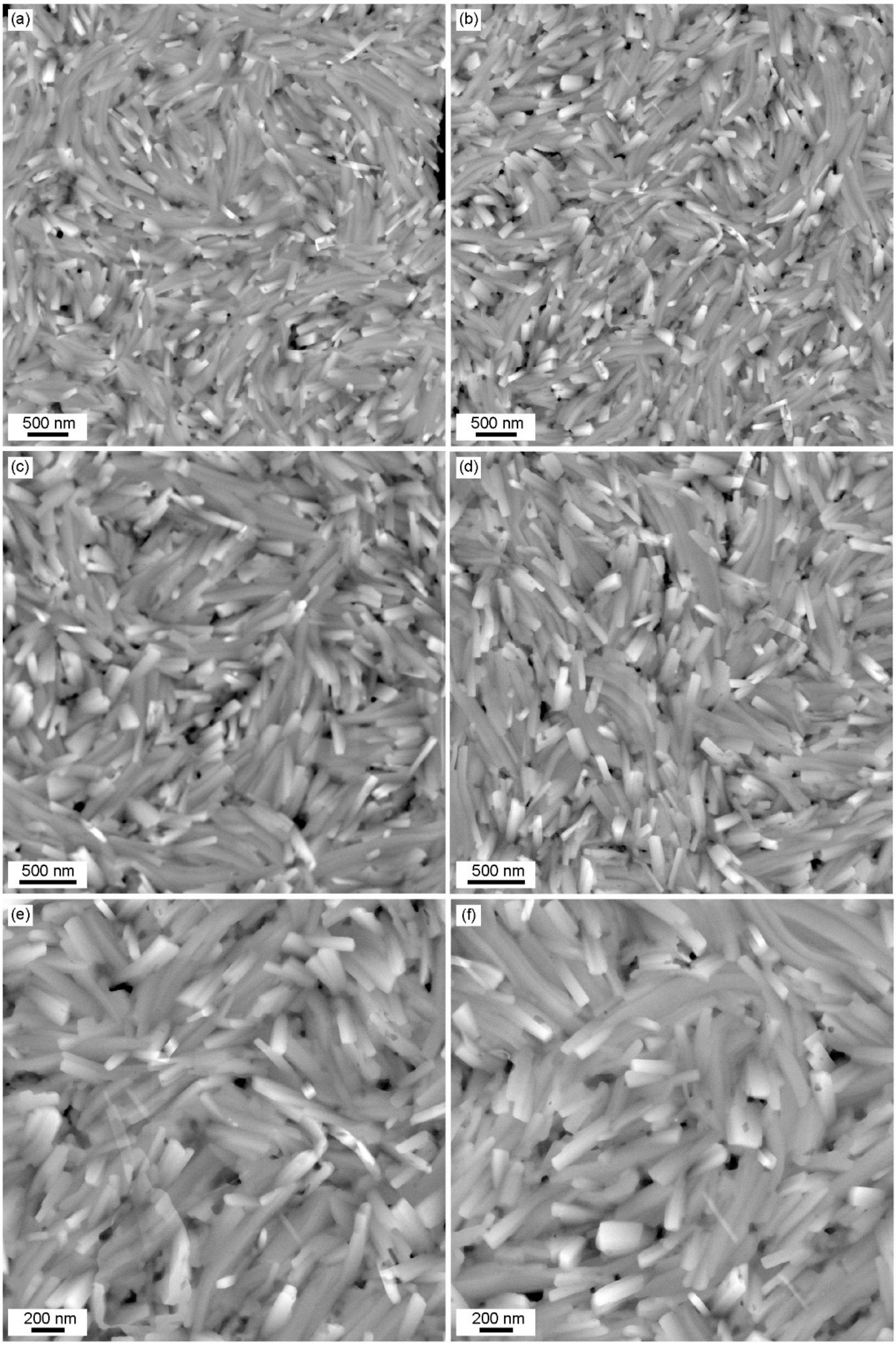

Figure S6. STEM images of a thin film of $[Ni(Hvanox)_2]$ deposited on a TEM grid using different magnifications (sample LTE-6d, thickness 180 nm).

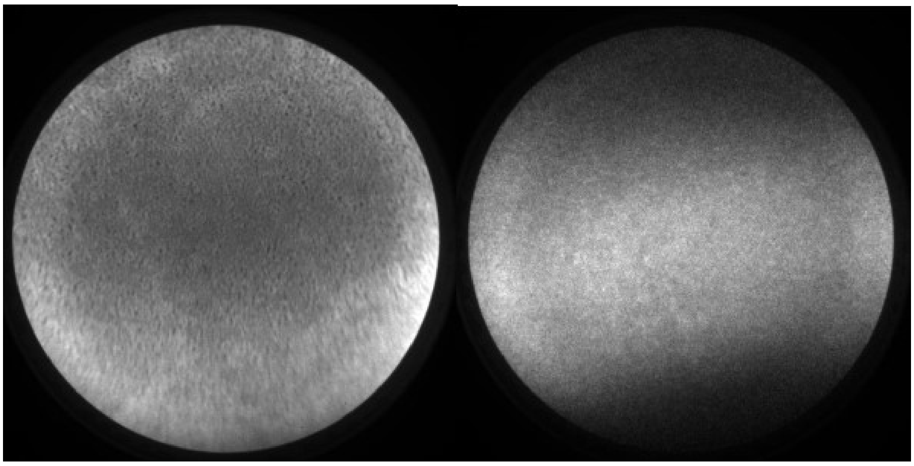

Figure S7. PEEM images of the center of the sample, field of view 102 µm, excited using a Hg-lamp ($h\nu$ = 5.2 eV, left) and by Al Kα ($h\nu$ = 1486.7 eV, right) show homogeneous coverage of the substrate with fine grains.

S3 References

1 B. Kumar, K. K. Prasad and S. K. Srivastawa, *Oriental Journal of Chemistry*, 2010, **26**, 1413–1418.
